# Longitudinal Associations between Anatomical Regions of Pain and Work Conditions: A Study from The SwePain Cohort

**DOI:** 10.3390/ijerph16122167

**Published:** 2019-06-19

**Authors:** Elena Dragioti, Björn Gerdle, Britt Larsson

**Affiliations:** Pain and Rehabilitation Centre and Department of Medical and Health Sciences, Linköping University, Linköping SE- 581 85, Sweden; bjorn.gerdle@liu.se (B.G.); britt.larsson@liu.se (B.L.)

**Keywords:** musculoskeletal pain, number of pain sites, cohort, physical workload, psychosocial work stressors, job strain

## Abstract

We investigated the time-based associations between workload (physical and mechanical), psychosocial work stressors (demands, control, and support), and the number of anatomical regions with pain (ARP). This population-based study with a two-year follow-up included 11,386 responders (5125 men, 6261 women; mean age: 48.8 years; SD: 18.5) living in south-eastern Sweden. Predictive associations were assessed through generalised linear models, and changes over time were examined using a generalised estimating equation. The results of both models were reported as parameter estimates (B) with 95% confidence interval (CIs). Mean changes in the number of ARP, workload, and psychosocial work stressors were stable over time. High mechanical workload and job demands were likely associated with the number of ARP at the two-year follow-up. In the reverse prospective model, we found that the number of ARP was also associated with high physical and mechanical workload and low job control and support. In the two time-based models of changes, we found a reciprocal association between number of ARP and mechanical workload. Our results add epidemiological evidence to the associations between work conditions and the extent of pain on the body. Components of work conditions, including job demands and mechanical strain, must be considered when organisations and health policy makers plan and employ ergonomic evaluations to minimise workplace hazards in the general population.

## 1. Introduction 

It is well established that musculoskeletal pain is a major public concern globally [1,2,3] and it is one of the leading causes of global years lived with disability [3]. Research has also shown that pain at more than one anatomical site is more frequent than pain at a single pain site [4,5,6,7] and results in high individual health consequences [8,9], poor quality of life [5,10], large societal costs [8], increased disability [9], and increased work disability [8,11,12,13]. However, most importantly, it has been noted that the magnitude of the association between musculoskeletal pain and negative health outcomes increases as the number of painful sites increases [8,12,13]. Consequently, assessing factors related to the number of anatomical regions with pain (ARP) is vital not only for optimum pain assessment but also for effective pain risk management. 

Emerging evidence suggests that work conditions such as workload (mechanical and physical) and psychosocial work stressors (e.g., high job demands and low support) are strongly linked with musculoskeletal pain [7,14,15,16,17,18,19,20,21,22,23]. The number of ARP has been related to physical workload [21], exposure to manual material handling, and awkward work postures [24]. Psychosocial work stress has been found to be an independent predictor of site-specific pain (i.e., neck/shoulder pain) [20]. High psychosocial demands, low job control, and low social support have been associated with prevalent pain at several body sites [14] as well as with an increase in pain regions [15,19]. In addition, high social support has been associated with lower likelihood of extended pain on the body [16].

However, most studies have focused on specific working populations [7,14,15,16,19,23] and relied on cross-sectional data [8,12,15,16,17,21,23]. A few prospective studies [18,19,25] have examined the extent to which the number of ARP contributes to work conditions and how the association between work conditions and the number of ARP develops over time, although these relationships have not been fully elucidated. For example, it is not known whether the number of ARP is an independent predictor of worse working conditions and whether changes in work conditions over time are associated with changes in the number of ARP or vice versa. In addition, no studies have fully addressed the time-dependent nature of potential confounders. 

This study explores associations between work conditions and the number of ARP in the general population considering three hypotheses over two years: (1) ARP is associated with baseline workload and psychosocial work stressors (prediction model 1); (2) workload and psychosocial work stressors are associated with baseline ARP (reverse prediction model 2); and (3) changes in ARP are associated with changes in workload and psychosocial work stressors over time (model of changes 1) and vice versa (reverse model of changes 2). In addition, this study examines the effect of age, gender, education, smoking, alcohol intake, psychical activity, distress, baseline physical workload, psychosocial work environment, and the number of ARP on the longitudinal associations between physical workload, psychosocial work environment, and the number of ARP.

## 2. Methods

**Figure 1:**
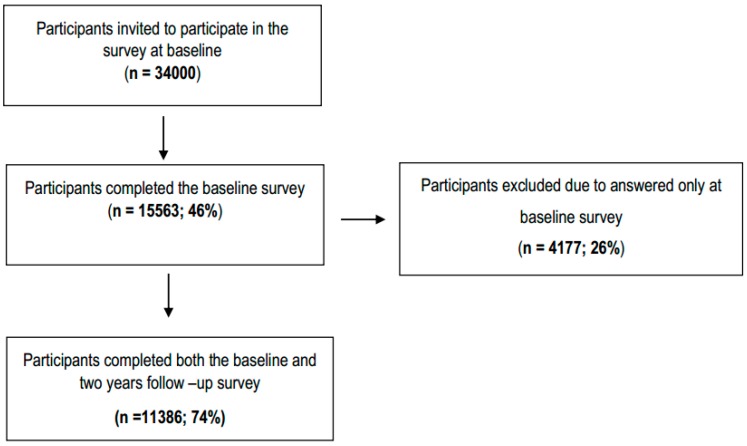
Flow chart outline the inclusion of participants for this study.

This prospective study, is a part of a large Swedish population-based cohort study (SwePain), assessing biopsychosocial aspects of pain from a sampling frame based on the Swedish Total Population Register [26,27]. The detailed methods and procedures have been described elsewhere [26,27]. Briefly, baseline data were collected using a representative stratified random sample of 34000 individuals from the general population in south-eastern Sweden. The collection of questionnaires ended in May 2013 and follow-up data were collected two years later. Only individuals who completed and returned the first questionnaire were eligible to participate in the follow-up assessment. Eligible individuals received a postal survey in March 2015, which could be returned by post or electronically, and two reminders were sent, if required. The collection of the follow-up data ended in May 2015 [27]. Both surveys at the two-time points included the same measurements. Inclusion criteria were women or men between 18 and 85 years old who had answered the survey at both time points (Figure 1). Moreover, according to instructions of the survey only individuals who were employed at baseline answered instruments of workload and psychosocial work stressors (for a description see below).

Completion of the survey was deemed to be patient informed consent. We used the Strengthening the Reporting of Observational Studies in Epidemiology (STROBE) statement for this study, as previously reported [27]. The study was approved by the local ethics committee of Linköping University, Sweden (Dnr: 2011 72/31). 

### 2.1. Outcomes

All outcomes were measured at baseline and at the two-year follow-up.

#### 2.1.1. The Number of Anatomical Regions with Pain (ARP)

We measured the number of ARP by counting the number of self-reported pain sites for the previous seven days on a body manikin divided into 45 sections on the front and on the back [28,29]. Based on these 45 sections, 23 separate ARP were defined as pain equally marked on the front and back of the manikin (e.g., shoulder and arm) or at least on one of the following: (1) foot right, (2) foot left, (3) lower leg right, (4) lower leg left, (5) thigh right, (6) thigh left, (7) hand right, (8) hand left, (9) forearm right, (10) forearm left, (11) upper arm right, (12) upper arm left, (13) shoulder right, (14) shoulder left, (15) neck/throat, (16) head right, (17) head left, (18) stomach, (19) chest, (20) gluteal, (21) low back, (22) upper back, and (23) genitals [5]. Next, we summed the total number of the 23 ARP to reflect the spatial distribution of pain on the body, ranging from 0 to 23. Higher scores indicate more pain on the body. In the literature, simple numerical pain measures have been widely used and can accurately classify and explain the extent of pain on the body in the general population better than more complicated measures [11,30].

#### 2.1.2. Workload 

We measured workload using the Swedish version of two workload indices: the mechanical exposure index (MEI) and physical exposure index (PHYI) [31]. The MEI consists of 11 items concerning work postures and movements, and the PHYI consists of seven items concerning work physical activity and lifting. Each item of the two indices was answered on a three-point scale: 1 = “not at all”; 2 = “somewhat”; and 3 = “a great deal.” For each scale, we summed the points (MEI ranging from 10 to 30 and PHYI ranging from 6 to 18). Higher scores indicate higher workload (mechanical and physical). Both indices are validated and widely used to assess the exposure of workload [22,23,31]. 

#### 2.1.3. Psychosocial Work Stressors

We assessed the psychosocial work stressors using the Job Content Questionnaire (JCQ) [32]. The JCQ consists of three subscales—job demands, job control, and job support [32]. The job demands subscale includes nine items concerning questions related to work strains and loads (e.g., working pace, hard work, excessive demands, time pressure, conflicting demands, and stressful work). The job control subscale includes nine items concerning decision latitude (e.g., influence at work and freedom to decide how work should be done) and skill discretion (e.g., development opportunities, skill, and creativity). Finally, the job support subscale includes eight items concerning support from management and co-workers. Each item was rated on a four-point scale (1 to 4) and the mean value in each subscale was calculated [32]. Higher scores indicate higher demands, better control, and better support. The JCQ is a validated and commonly used instrument [15,16,23,32]. 

### 2.2. Exposure Variables 

All outcomes (i.e., ARP, MEI, PHYI, and JCQ) served also as exposure variables and were measured at baseline and the two-year follow-up.

### 2.3. Potential Confounders

We selected seven covariates as potential confounders known to affect both the number of ARP and work conditions: age, gender (women/men), education (university/other), smoking (smoker/ non-smoker), alcohol intake (yes/no), psychical activity (frequency and intensity), and distress [33,34]. The assessment of smoking status was based on a single question: Do you smoke cigarettes/cigarillos (Yes/No)? The assessment of alcohol intake was also based on a single question: Do you drink alcohol (Yes/No)? We used the Godin Leisure-Time Exercise Questionnaire (GLTE) to assess psychical activity [35]. The GLTE asks four questions to assess how many times (per week) and how intensely (strenuous, moderate, and mild) the respondent exercises. The different intensities are described with examples. A total physical activity per week score is calculated using the times per week for the different intensities: 9 for strenuous, 5 for moderate, and 3 for mild [36]. A high score indicates higher intensity and higher frequency of weekly leisure-time activities. The distress was measured using the General Well Being Scale (GWBS) [37]. The GWBS includes 18 items that yield a total score ranging from 0 to 110 (high scores indicate positive well-being and low scores indicate distress). The first 14 questions use a six-point rating scale (ranging from 0 to 5) and the remaining four items use an 11-point rating scale with the end-points 0 (very concerned) and 10 (not concerned at all). We also measured all potential confounders at baseline and the two-year follow-up.

### 2.4. Statistical Analysis

**Figure 2:**
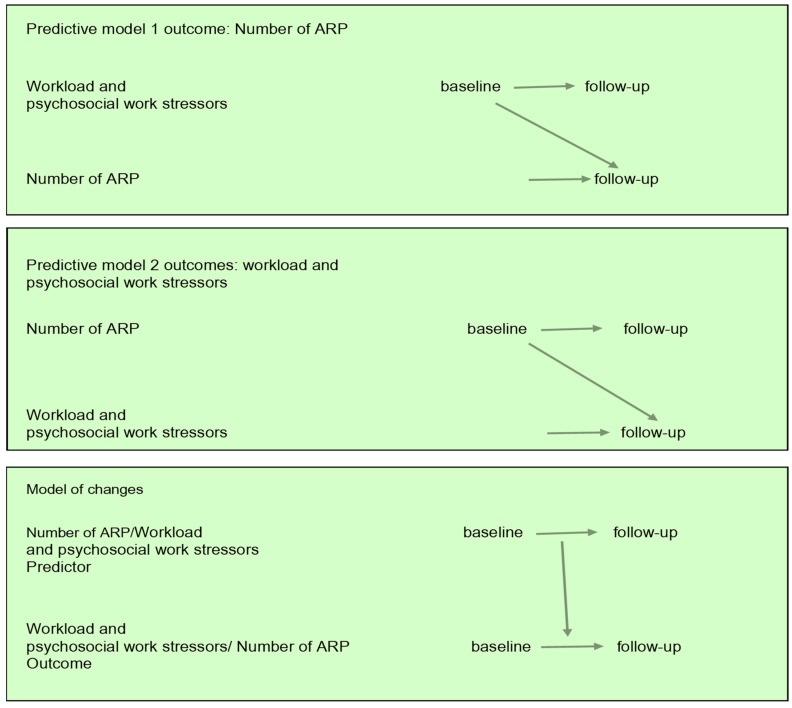
Graphic depiction of the models used to examine the longitudinal association between the number of anatomical pain regions, workload, and psychosocial work stressors. ARP = anatomical regions with pain.

Data were analysed using SPSS version 25.0 for Windows (IBM Corporation, Armonk, NY, USA). All tests were two-tailed and *p* < 0.05 was regarded as significant. We presented all the variables of interest using descriptive statistics: mean ± standard deviation (SD) for continuous variables and *n* (%) for categorical variables. To study the first two hypotheses, we used a series of generalised linear models (GLM). GLM is a flexible generalisation of ordinary regression analyses that allow for response variables that have error distribution models other than a normal distribution [38]. The data on the ARP and work conditions served as linear-response data, so the identity link function was used with a maximum likelihood estimation (MLE) and robust standard errors. We present the GLM results as parameter estimates (B) with 95% confidence interval (CIs). We used two models to assess the prediction hypotheses (Figure 2). The first model addressed the question of whether baseline work conditions predict the number of ARP at follow-up (prediction model 1). The second model, an inverse of the first model, addressed the question of whether the baseline number of ARP predicts work conditions at follow-up (reverse prediction model 2) (Figure 2). 

To study the third hypothesis (models of changes), we used the Generalized Estimated Equations (GEEs) with robust standard errors to examine the longitudinal associations between ARP and work conditions over time. Therefore, the models of change also had two forms, either changes in the number of ARP (model of changes 1) or changes in work conditions as outcome variables (reverse model of changes 2) (Figure 2). GEE, which considers dependency between repeated measures [39], is a flexible method for longitudinal analysis and can be used to analyse correlated data with binary, discrete, or continuous outcomes [39]. This technique allows all participants to be included in the analysis even when data are missing. We used GEEs with a linear distribution and an identity link function while we employed an independent working correlation matrix. We presented the results as parameter estimates (B) with 95% confidence interval (CIs). Lastly, when we found significant associations between ARP and work conditions in the models of changes, we performed a multiple post-hoc sensitivity analysis to show how the interaction between these variables depend on potential effects of age, gender, education, smoking, alcohol intake, psychical activity, and distress (i.e., weaker vs. stronger). 

In all analyses, we present the crude versus adjusted parameter estimates. Using the Wald test, we tested the statistical significance of both GLM and GEE models. To estimate the multicollinearity among covariates, we used the variance inflation factor (VIF) with a cut-off score of >2 as an indicator of multicollinearity [40]. As the VIF was <2, all covariates were included in the analysis. We tested for unequal possibilities of sample selection by weighting cases regarding age strata, gender, and city in all models. Statistics Sweden calculated the sampling weights. A stratified analysis by age and gender was also performed in all models. 

## 3. Results

### 3.1. Population Characteristics

The flowchart of the sample choice is shown in Figure 1. At baseline, 15,563 individuals (54% women) completed and returned the questionnaire, a response rate of 46%. Of these, 4177 were excluded because they did not take part in the follow-up (26%). Thus, the final sample consisted of 11,386 responders (74% response rate; 55% women). The dropout analysis showed that the response rate at follow-up was lower for younger ages, men, singles, and those without a university education [27]. Table 1 shows the population general characteristics for both time points. The mean age at baseline was 48.8 (SD = 18.5). The number of ARP, workload, and psychosocial work stressors were stable over time (Table 2).

### 3.2. Prediction Models 

The results of the GLM (crude, baseline adjusted, and fully adjusted) analyses are presented in Table 3. The baseline adjusted models of the prediction model 1 (Table 3, upper), evaluating the number of ARP at follow-up in relation to baseline workload and psychosocial work stressors, showed significant correlations between baseline MEI, psychosocial work stressors, and number of ARP at follow-up (all *p* < 0.001). 

The fully-adjusted models (Table 3, upper) revealed that MEI and job demands predicted the number of ARP two years later. The positive parameter estimates showed that higher baseline job demands and mechanical exposure were associated with higher number of ARP (i.e., pain over more parts of the body) (Table 3, upper). 

When we evaluated work conditions at follow-up in relation to baseline number of ARP, we found that the number of APR predicted the investigated workload and psychosocial work stressors (all *p* < 0.001) according to the baseline adjusted models of the reverse prediction model 2 (Table 3, lower). 

All of these associations—except the prediction of job demands (*p* = 0.829)—remained significant in the fully adjusted models (Table 3, lower; all *p* < 0.02). The positive parameter estimates in these predictions showed that a higher number of ARP (i.e., pain on more areas of the body) was associated with higher MEI and PHYI. The negative parameter estimates revealed that higher number of ARP was associated with lower job control and job support. Table 4 and Table 5 present the results of stratified analysis by age and gender. 

### 3.3. Models of Changes 

The results of the GEE (crude, baseline adjusted, and fully adjusted) analyses of changes over time are presented in Table 6. According to the baseline adjusted models, all changes in workload and psychosocial work stressor variables investigated showed significant associations with changes in the number of ARP (Table 6, upper; all *p* ≤ 0.006) and vice versa (Table 6, lower; *p* ≤ 0.002). In the fully adjusted models (i.e., adjustments of changes in the non-time-dependent gender, and in the time-dependent number of ARP, workload, and psychosocial work stressors, and the other potential confounders), there were significant associations between changes in the number of ARP and MEI over time (Table 6, upper part) and vice versa (Table 6, lower; all *p* < 0.001) with similar strength of associations. The positive parameter estimates indicated that higher number of ARP was associated with higher MEI and vice versa. This was not the case for the PHYI and the psychosocial work stressors (Table 6), where a non-significant association between changes in the number of ARP, PHYI, job demands, job control, and job support were found (*p* > 0.05). 

In the fully adjusted model of changes using the number of ARP as outcome (model of changes 1) (Table 6, upper), the analysis showed the associations between changes in the number of ARP and MEI are modified by age (*p* < 0.001), gender (*p* < 0.001), physical activity (*p* < 0.001), and distress (*p* < 0.001). Hence, changes in the number of ARP and MEI are weaker in younger people and in people with high physical activity than in older people and people with lower physical activity. However, the association between changes in the number of ARP and MEI were stronger in women and in people with elevated levels of distress than in men and people with low levels of distress. 

In the reverse fully adjusted model of changes (reverse model of changes 2) (Table 6, lower), which uses changes in workload and psychosocial work stressors as outcomes, the same statistically significant interactions were observed. Tables S2 and S3 present the results of stratified analysis by age and gender. 

## 4. Discussion 

We used predictive and change models (time-based) and their reverse models to examine all longitudinal associations between the number of ARP and work conditions in a large sample of the general population. According to the fully adjusted models, mechanical exposure (MEI) and job demands were significant predictors of the number of ARP at follow-up, while the number of ARP significantly predicted physical (PHYI) and mechanical exposure (MEI) and low job control and support at follow-up. Therefore, these results do not support our first two hypotheses: the predictive associations between workload, psychosocial work stressors, and the number of ARP would be similar, showing a reciprocal association. After adjusting for changes in time-depended confounders in the fully adjusted models, the model reflecting changes suggests that only the number of ARP and mechanical exposure were positively associated over time. Hence, this finding partly supports our third hypothesis: there is a direct time-based association between changes in both factors with an increase in the number of ARP followed by an increase in mechanical exposure and vice versa. 

The baseline adjusted models (Table 3 and Table 6) showed significant associations for most analyses. Only a few of these remained significant in the fully adjusted models, which are discussed below. As this is a population study, the characteristics of the subjects are more heterogenous than in studies investigating a certain workplace or profession. Therefore, we adjusted for multiple aspects. 

The association between work conditions, such as high mechanical exposure and job demands, and an increasing number of ARP were expected in both predictive and change models. Hence, our results are in line with the few prospective studies showing an association between high mechanical exposure, high job demands, and increased risk of musculoskeletal pain [18,19,25]. For example, in a longitudinal study of 5136 employees from the Netherlands, Oakman et al. [19] found that high job demands increased the risk of musculoskeletal pain by almost 60% during a four-year measurement period. Our findings are also consistent with several cross-sectional studies related to this field [7,12,15,16,17,21,23]. 

In the first model of changes (Table 6, upper), however, we found that only mechanical exposure change was associated with change in number of ARP. This discrepancy between the first prediction model (Table 3, upper) and the first model of changes (Table 6, upper) may indicate that individual and workplace characteristics were experienced differently over time. This hypothesis should be examined in future research. Additionally, in contrast with previous studies, we did not confirm an association between low job control and low job support with ARP [14,15]. However, it is important to note that our results cannot be directly compared to the other studies due to different pain assessments and definitions. In addition, our study is a population study whereas several other studies focus on more selected cohorts (e.g., health care providers and police.)

The confirmation of our hypothesis that the number of ARP (also in both reverse models) was predictive of high mechanical and physical exposure, low job control, and low support is somewhat surprising and novel. However, the magnitudes of the observed associations were low (b = −0.01 to 0.03) in the reserve prediction model, but higher in the reverse model of changes (b = 0.10). We cannot present any strong explanation for this intriguing finding apart from the speculation that people with more extent of pain on the body or despite their pain may continue to work under physical and psychosocial work hazards. It is also possible that due to pain people have more difficulty in performing the tasks and therefore will perceive mechanical, physical, and psychosocial work factors as more difficult than they are in reality and thus a vicious circle has arisen. It is also possible that people with more extent of pain may be reluctant to report their pain status because pain itself may make them feel ashamed. Emerging evidence shows that the feelings of shame and isolation stand as the two main unmet psychological needs in people with pain [41,42]. This also may mean that the work assessments used in this study are not performed or are not able to capture all workplace hazards. Furthermore, such time-based reciprocal effects have been reported for the association between low back pain and depressive symptoms [43]. In any case, these findings should be treated with caution and should be investigated in future prospective studies. 

Unsurprisingly, the longitudinal association between changes in the number of ARP and mechanical exposure seems to be partly conditioned by changes in other factors, as we found that the association was stronger in women and in the severely distressed, indicating that these factors would strengthen the association between the number of ARP and mechanical exposure. This association was weaker in younger people and in people with high physical activity, indicating that these factors would weaken this association over time. These results are consistent with other studies that show that women and distressed patients are more vulnerable to musculoskeletal pain [11,25,44], whereas younger people and people who exercise are less vulnerable to musculoskeletal pain [45,46].

To our knowledge, this longitudinal study is the first attempt to examine the temporal association between work conditions and the number of ARP after adjusting for several potential confounders (i.e., the fully adjusted models). We examined this association in a large population-based cohort with measurements at two time points within a period of two years. Therefore, this study adds epidemiological evidence on how work conditions and the number of ARP develop and associate over time in a general population. In addition, the repeated measurement of all the studied confounders allowed us to examine the time-dependent effect of these factors on the observed associations between work conditions and the number of ARP. Moreover, baseline adjusted models tend to overestimate the relationships without requiring the fully adjusted models especially when used in a population study.

We are aware that our research may have two major limitations. The first is that our results mostly rely on self-report measurements and postal surveys, so we cannot exclude the possibility of an overestimation of our results. However, both methods of measurements are common in epidemiological research [10,15,19,25], and we simultaneously used two of the most suitable working models of workload [22,23,31] and psychosocial work stressors [15,16,23,32]. The second limitation is that we did not adjust for employment status or history (e.g., current employment vs. previous employment); such adjustments should be considered in future studies for a more accurate precision of estimates because information on employees’ work histories and sorting for it could address the problem that employees are not randomly assigned into workplaces [47]. Furthermore, one may argue that our pain assessment did not consider the spatial distribution of pain—i.e., pain in anatomical regions that are related to each other; however, the simple counting method applied here has been proven powerful [11,30]. 

## 5. Conclusions

Work conditions are crucial factors to consider when assessing factors related to the number of pain regions. Our fully adjusted models show that both high mechanical workload and job demands were associated with the number of ARP at the two-year follow-up. This was not the case for the other work-related factors such as physical workload, job control, and job support. In reverse, a prospective association was observed between the number of ARP, high workload (both mechanical and physical), and low job control. This study also highlights that changes in mechanical workload and the number of ARP are reciprocally and directly associated over time. Our results, therefore, add epidemiological evidence to a reciprocal association between mechanical workload and the number of ARP over time. The unfavourable effect of this association was more prominent in women and severely distressed individuals and less prominent in younger people and people who participate in regular physical exercise. Work conditions, including job demands and mechanical strain, must be considered when organisations and health policy makers plan and employ ergonomic evaluations to minimise workplace hazards such as pain aspects. Moreover, the implementation of workplace interventions such as ergonomic interventions, education intervention, biofeedback training, and job stress management training should be also considered to minimise workplace hazards in relation to pain. Evidence shows that such interventions have proven useful in the prevention of musculoskeletal risk in working populations [48,49,50]. Our results may add aspects important to consider when managing the risk for deterioration of pain (e.g., pain spreading) and the results need to be validated by future longitudinal studies. 

## Figures and Tables

**Table 1:** Population characteristics at baseline and two-year follow-up.

Characteristic; Mean (SD), Unless Otherwise Stated	Baseline	Follow-Up
Age	48.8 (18.5)	50.8 (18.5)
Women; *n* (%)	6261 (55.0%)	6261 (55.0%)
University; *n* (%)	4307 (38.4%)	4414 (39.5%)
Smokers; *n* (%)	1315 (11.8%)	1127 (10.2%)
Alcohol intake; *n* (%)	9021 (80.1%)	7355 (82.3%)
Psychical activity (GLTEQ index; range 0–194)	33.6 (25.6)	34.3 (26.0)
Distress (GWBS; range; 0–110)	76.2 (18.1)	75.9 (18.3)

GLTE = Godin Leisure-Time Exercise Questionnaire; GWBS = General Well-Being Schedule; SD = standard deviation. Note: Higher scores on the psychical activity scale indicate more psychical activity, whereas higher scores on the distress scale indicate less distress.

**Table 2:** Number of anatomical pain regions, workload, and psychosocial work stressors over time.

Variables	Baseline		Follow-Up	
	*n*	Mean (SD)	*n*	Mean (SD)
Number of anatomical regions with pain (ARP; range 0–23)	11,386	1.7 (2.8)	11,386	1.8 (2.8)
**Workload**				
Mechanical exposure index (MEI; range 10–30)	9140	15.1 (4.0)	9121	14.9 (4.1)
Physical exposure index (PHYI; range 6–18)	9544	9.8 (3.0)	9486	9.7 (3.1)
**Psychosocial work stressors**				
Job demands (mean JCQ; range 1–4)	10,376	2.5 (0.6)	10,294	2.5 (0.6)
Job control (mean JCQ; range 1–4)	10,388	3.0 (0.5)	10,299	3.0 (0.5)
Job support (mean JCQ; range 1–4)	10,281	3.2 (0.5)	10,237	3.2 (0.5)

ARP = anatomical regions with pain; JCQ = Job Content Questionnaire; MEI = Mechanical exposure index; PHYI = Physical exposure index; SD = standard deviation; *n* = number of answers. Note: Higher scores on the scales indicate more extent of pain on the body, high workload, high demands, better control, and better support.

**Table 3:** Prediction models between workload, psychosocial work stressors, and the number of anatomical pain regions.

Outcome and Exposure Variables	Crude Models		Baseline Adjusted Models *		Fully Adjusted Models **	
	B (95% CI)	*p*-Value	B (95% CI)	*p*-Value	B (95% CI)	*p*-Value
**Prediction model 1, outcome: number of ARP at follow-up**						
Predictor: baseline mechanical exposure index	0.14 (0.13 to 0.14)	<0.001	0.03 (0.02 to 0.03)	<0.001	0.03 (0.02 to 0.03)	<0.001
Predictor: baseline physical exposure index	0.11 (0.10 to 0.12)	<0.001	0.01 (−0.02 to 0.03)	0.618	0.00 (−0.01 to 0.01)	0.826
Predictor: baseline job demands	0.64 (0.62 to 0.66)	<0.001	0.17 (0.15 to 0.19)	<0.001	0.13 (0.10 to 0.15)	<0.001
Predictor: baseline job control	−0.50 (−0.52 to −0.48)	<0.001	−0.14 (−0.16 to −0.12)	<0.001	−0.05 (−0.16 to 0.08)	0.502
Predictor: baseline job support	−0.63 (−0.65 to −0.60)	<0.001	−0.07 (−0.08 to −0.05)	<0.001	−0.04 (−0.15 to 0.07)	0.520
**Prediction model 2, outcomes: workload and psychosocial work stressors at follow-up**						
***Prediction model 2, outcome: mechanical exposure index at follow-up***						
Predictor: baseline number of ARP	0.28 (0.27 to 0.29)	<0.001	0.04 (0.04 to 0.05)	<0.001	0.03 (0.02 to 0.04)	<0.001
***Prediction model 2, outcome: physical exposure index at follow-up***						
Predictor: baseline number of ARP	0.11 (0.11 to 0.12)	<0.001	0.01 (0.00 to 0.01)	<0.001	0.02 (0.00 to 0.01)	0.018
***Prediction model 2, outcome: job demands at follow-up***						
Predictor: baseline number of ARP	0.03 (0.02 to 0.03)	<0.001	0.01 (0.00 to 0.01)	<0.001	0.00 (−0.00 to 0.00)	0.829
***Prediction model 2, outcome: job control at follow-up***						
Predictor: baseline number of ARP	−0.01 (−0.01 to −0.01)	<0.001	−0.01 (−0.01 to −0.01)	<0.001	−0.01 (−0.01 to −0.00)	<0.001
***Prediction model 2, outcome: job support at follow-up***						
Predictor: baseline number of ARP	−0.03 (−0.03 to −0.03)	<0.001	−0.01 (−0.01 to −0.01)	<0.001	−0.01 (−0.01 to −0.01)	<0.001

ARP = anatomical regions with pain; B = regression coefficients; CI = confidence intervals. * Model adjusted for baseline physical workload, psychosocial work environment, and number of ARP; ** Model adjusted for baseline age, gender, education, smoking, alcohol intake, psychical activity, distress, and baseline workload, psychosocial work stressors, and the number of ARP.

**Table 4:** Prediction models between workload, psychosocial work stressors, and the number of anatomical pain regions stratified by baseline age.

Outcome and Exposure Variables	Crude Models		Baseline Adjusted Models *		Fully Adjusted Models **	
	B (95% CI)	*p*-Value	B (95% CI)	*p*-Value	B (95% CI)	*p*-Value
**Age < 50 Years**						
**Prediction model 1, outcome: number of ARP at follow-up**						
Predictor: baseline mechanical exposure index	0.12 (0.12 to 0.13)	<0.001	0.03 (0.02 to 0.04)	<0.001	0.03 (0.03 to 0.04)	<0.001
Predictor: baseline physical exposure index	0.10 (0.10 to 0.11)	<0.001	−0.01 (−0.01 to 0.01)	0.096	0.00 (−0.01 to 0.01)	0.627
Predictor: baseline job demands	0.61 (0.58 to 0.64)	<0.001	0.23 (0.21 to 0.25)	<0.001	0.18 (0.16 to 0.20)	<0.001
Predictor: baseline job control	−0.35 (−0.38 to −0.32)	<0.001	−0.13 (−0.15 to −0.10)	<0.001	−0.05 (−0.16 to 0.08)	0.502
Predictor: baseline job support	−0.45 (−0.48 to −0.43)	<0.001	0.03 (0.01 to 0.05)	0.041	−0.04 (−0.15 to 0.07)	0.520
**Age ≥ 50 years**						
**Prediction model 1, outcome: number of ARP at follow-up**						
Predictor: baseline mechanical exposure index	0.15 (0.14 to 0.15)	<0.001	0.02 (0.02 to 0.03)	<0.001	0.01 (0.02 to 0.03)	0.008
Predictor: baseline physical exposure index	0.13 (0.12 to 0.13)	<0.001	0.01 (0.00 to 0.02)	0.009	0.03 (0.02 to 0.03)	<0.001
Predictor: baseline job demands	0.70 (0.67 to 0.72)	<0.001	0.11 (0.08 to 0.13)	<0.001	0.04 (0.01 to 0.07)	0.024
Predictor: baseline job control	−0.78 (−0.82 to −0.74)	<0.001	−0.20 (−0.23 to −0.17)	<0.001	0.01 (−0.03 to 0.05)	0.571
Predictor: baseline job support	−0.85 (−0.89 to −0.82)	<0.001	−0.18 (−0.21 to −0.15)	<0.001	−0.07 (−0.10 to −0.03)	<0.001
**Age < 50 years**						
***Prediction model 2, outcome: mechanical exposure index at follow-up***						
Predictor: baseline number of ARP	0.26 (0.25 to 0.27)	<0.001	0.06 (0.05 to 0.07)	<0.001	0.03 (0.02 to 0.03)	<0.001
***Prediction model 2, outcome: physical exposure index at follow-up***						
Predictor: baseline number of ARP	0.10 (0.09 to 0.11)	<0.001	−0.01 (−0.01 to −0.00)	0.001	−0.02 (−0.02 to −0.01)	<0.001
***Prediction model 2, outcome: job demands at follow-up***						
Predictor: baseline number of ARP	0.03 (0.02 to 0.03)	<0.001	0.01 (0.00 to 0.01)	<0.001	0.00 (−0.00 to 0.00)	0.093
***Prediction model 2, outcome: job control at follow-up***						
Predictor: baseline number of ARP	−0.01 (−0.01 to −0.01)	<0.001	0.00 (0.00 to 0.00)	0.168	0.01 (0.01 to 0.01)	<0.001
***Prediction model 2, outcome: job support at follow-up***						
Predictor: baseline number of ARP	−0.03 (−0.03 to −0.03)	<0.001	−0.02 (−0.02 to −0.01)	<0.001	−0.01 (−0.01 to −0.01)	<0.001
**Age ≥ 50 years**						
**Prediction model 2, outcomes: workload and psychosocial work stressors at follow-up**						
***Prediction model 2, outcome: mechanical exposure index at follow-up***						
Predictor: baseline number of ARP	0.30 (0.29 to 0.32)	<0.001	0.02 (0.01 to 0.03)	<0.001	0.03 (0.02 to 0.04)	<0.001
***Prediction model 2, outcome: physical exposure index at follow-up***						
Predictor: baseline number of ARP	0.13 (0.12 to 0.14)	<0.001	−0.01 (−0.01 to −0.00)	0.003	0.00 (−0.00 to 0.01)	0.232
***Prediction model 2, outcome: job demands at follow-up***						
Predictor: baseline number of ARP	0.03 (0.02 to 0.03)	<0.001	0.01 (0.00 to 0.01)	<0.001	0.01 (0.00 to 0.01)	<0.001
***Prediction model 2, outcome: job control at follow-up***						
Predictor: baseline number of ARP	−0.02 (−0.02 to −0.01)	<0.001	−0.01 (−0.01 to −0.01)	<0.001	0.01 (0.00 to 0.01)	0.004
***Prediction model 2, outcome: job support at follow-up***						
Predictor: baseline number of ARP	−0.03 (−0.03 to −0.03)	<0.001	−0.01 (−0.01 to −0.01)	<0.001	−0.01 (−0.01 to −0.01)	<0.001

ARP = anatomical regions with pain; B = regression coefficients; CI = confidence intervals. * Model adjusted for baseline physical workload, psychosocial work environment, and number of ARP; ** Model adjusted for baseline gender, education, smoking, alcohol intake, psychical activity, distress, and baseline workload, psychosocial work stressors, and the number of ARP.

**Table 5:** Prediction models between workload, psychosocial work stressors, and the number of anatomical pain regions stratified by baseline gender.

Outcome and Exposure Variables	Crude Models		Baseline Adjusted Models *		Fully Adjusted Models **	
	B (95% CI)	*p*-Value	B (95% CI)	*p*-Value	B (95% CI)	*p*-Value
**Men**						
**Prediction model 1, outcome: number of ARP at follow-up**						
Predictor: baseline mechanical exposure index	0.10 (0.09 to 0.13)	<0.001	0.02 (0.02 to 0.03)	<0.001	0.02 (0.01 to 0.03)	<0.001
Predictor: baseline physical exposure index	0.12 (0.10 to 0.14)	<0.001	0.01 (−0.03 to 0.04)	0.782	0.01 (−0.03 to 0.05)	0.603
Predictor: baseline job demands	0.55 (0.43 to 0.66)	<0.001	0.18 (0.16 to 0.19)	<0.001	0.11 (0.09 to 0.13)	<0.001
Predictor: baseline job control	−0.25 (−0.39 to −0.11)	<0.001	−0.06 (−0.08 to 0.04)	<0.001	−0.07 (−0.09 to −0.03)	<0.001
Predictor: baseline job support	−0.56 (−0.69 to −0.45)	<0.001	−0.13 (−0.16 to −0.11)	<0.001	−0.02 (−0.04 to 0.01)	0.180
**Women**						
**Prediction model 1, outcome: number of ARP at follow-up**						
Predictor: baseline mechanical exposure index	0.19 (0.16 to 0.22)	<0.001	0.03 (0.03 to 0.04)	<0.001	0.13 (0.08 to 0.17)	<0.001
Predictor: baseline physical exposure index	0.14 (0.10 to 0.17)	<0.001	0.01 (0.00 to 0.02)	0.010	−0.01 (−0.07 to 0.05)	0.680
Predictor: baseline job demands	0.65 (0.48 to 0.81)	<0.001	0.26 (0.07 to 0.45)	0.007	0.12 (0.09 to 0.15)	<0.001
Predictor: baseline job control	−0.47 (−0.67 to −0.27)	<0.001	−0.25 (−0.48 to −0.01)	0.044	0.10 (0.06 to 0.14)	<0.001
Predictor: baseline job support	−0.63 (−0.82 to −0.43)	<0.001	−0.34 (−0.56 to −0.11)	0.004	0.12 (0.09 to 0.16)	<0.001
**Men**						
**Prediction model 2, outcomes: workload and psychosocial work stressors at follow-up**						
***Prediction model 2, outcome: mechanical exposure index at follow-up***						
Predictor: baseline number of ARP	0.42 (0.41 to 0.44)	<0.001	0.02 (0.01 to 0.03)	<0.001	0.01 (−0.01 to 0.02)	0.284
***Prediction model 2, outcome: physical exposure index at follow-up***						
Predictor: baseline number of ARP	0.23 (0.22 to 0.24)	<0.001	0.02 (0.00 to 0.01)	<0.001	0.02 (0.01 to 0.03)	<0.001
***Prediction model 2, outcome: job demands at follow-up***						
Predictor: baseline number of ARP	0.03 (0.03 to 0.04)	<0.001	0.01 (0.00 to 0.01)	0.029	−0.01 (−0.01 to −0.00)	0.002
***Prediction model 2, outcome: job control at follow-up***						
Predictor: baseline number of ARP	−0.01 (−0.01 to −0.00)	<0.001	−0.01 (−0.01 to −0.00)	0.042	0.01 (0.00 to 0.01)	<0.001
***Prediction model 2, outcome: job support at follow-up***						
Predictor: baseline number of ARP	−0.03 (−0.04 to −0.03)	<0.001	−0.01 (−0.01 to −0.01)	<0.001	−0.01 (−0.01 to −0.01)	0.013
**Women**						
**Prediction model 2, outcomes: workload and psychosocial work stressors at follow-up**						
***Prediction model 2, outcome: mechanical exposure index at follow-up***						
Predictor: baseline number of ARP	0.23 (0.22 to 0.24)	<0.001	0.05 (0.05 to 0.06)	<0.001	0.05 (0.04 to 0.05)	<0.001
***Prediction model 2, outcome: physical exposure index at follow-up***						
Predictor: baseline number of ARP	0.09 (0.08 to 0.09)	<0.001	−0.01 (−0.02 to 0.00)	<0.001	−0.01 (−0.02 to 0.00)	<0.001
***Prediction model 2, outcome: job demands at follow-up***						
Predictor: baseline number of ARP	0.02 (0.02 to 0.02)	<0.001	0.01 (0.00 to 0.01)	<0.001	0.01 (0.00 to 0.01)	<0.001
***Prediction model 2, outcome: job control at follow-up***						
Predictor: baseline number of ARP	−0.01 (−0.01 to −0.01)	<0.001	−0.00 (−0.01 to 0.00)	0.158	0.01 (0.00 to 0.01)	<0.001
***Prediction model 2, outcome: job support at follow-up***						
Predictor: baseline number of ARP	−0.03 (−0.03 to −0.02)	<0.001	−0.01 (−0.01 to −0.01)	<0.001	−0.01 (−0.01 to −0.01)	<0.001

ARP = anatomical regions with pain; B = regression coefficients; CI = confidence intervals. * Model adjusted for baseline physical workload, psychosocial work environment, and number of ARP; ** Model adjusted for baseline age, education, smoking, alcohol intake, psychical activity, distress, and baseline workload, psychosocial work stressors, and the number of ARP.

**Table 6:** Model of changes over time between workload, psychosocial work stressors, and the number of anatomical pain regions.

Outcome and Exposure Variables	Crude Models		Baseline Adjusted Models *		Fully Adjusted Models **	
	B (95% CI)	*p*-Value	B (95% CI)	*p*-Value	B (95% CI)	*p*-Value
**Model of change 1, outcome: change in the number of ARP**						
Predictor: change in mechanical exposure index	0.12 (0.10 to 0.13)	<0.001	0.12 (0.10 to 0.13)	<0.001	0.10 (0.08 to 0.12)	<0.001
Predictor: change in physical exposure index	0.09 (0.07 to 0.10)	<0.001	−0.03 (−0.05 to −0.01)	0.006	−0.00 (−0.00 to 0.00)	0.982
Predictor: change in job demands	0.39 (0.36 to 0.46)	<0.001	0.19 (0.11 to 0.27)	<0.001	−0.01 (−0.10 to 0.01)	0.815
Predictor: change in job control	−0.38 (−0.47 to −0.29)	<0.001	−0.18 (−0.28 to −0.08)	<0.001	0.01 (−0.10 to 0.12)	0.877
Predictor: change in job support	−0.48 (−0.44 to −0.38)	<0.001	−0.33 (−0.43 to −0.23)	<0.001	−0.07 (−0.17 to 0.01)	0.228
**Model of change 2, outcomes: workload and psychosocial work stressors**						
***Model of change 2, outcome: change in mechanical exposure index***						
Predictor: change in the number of ARP	0.20 (0.17 to 0.22)	<0.001	0.13 (0.11 to 0.15)	<0.001	0.12 (0.10 to 0.14)	<0.001
***Model of change 2, outcome: change in physical exposure index***						
Predictor: change in the number of ARP	0.08 (0.06 to 0.09)	<0.001	−0.02 (−0.01 to −0.00)	0.002	0.00 (−0.02 to 0.02)	0.987
***Model of change 2, outcome: change in job demands***						
Predictor: change in the number of ARP	0.02 (0.02 to 0.03)	<0.001	0.01 (0.00 to 0.02)	<0.001	−0.01 (−0.01 to 0.01)	0.810
***Model of change 2, outcome: change in job control***						
Predictor: change in the number of ARP	−0.01 (−0.02 to −0.01)	<0.001	−0.01 (−0.01 to −0.01)	<0.001	−0.00 (−0.00 to 0.00)	0.998
***Model of change 2, outcome: change in job support***						
Predictor: change in the number of ARP	−0.02 (−0.03 to −0.02)	<0.001	−0.01 (−0.02 to −0.01)	<0.001	−0.01 (−0.01 to 0.01)	0.249

ARP = anatomical regions with pain; B = regression coefficients; CI = confidence intervals. * Model adjusted for changes in workload, psychosocial work stressors, and number of ARP; ** Model adjusted for time-independent gender, and time-depended changes in age, education, smoking, alcohol intake, psychical activity, distress, and changes in workload, psychosocial work stressors, and the number of ARP.

## Supplementary Materials

Supplementary materials can be accessed at https://www.mdpi.com/1660-4601/16/12/2167/s1. Table S1: Strengthening the Reporting of Observational Studies in Epidemiology (STROBE) statement checklist, Table S2: Model of changes over time between workload, psychosocial work stressors, and the number of anatomical pain regions by age, Table S3: Model of changes over time between workload, psychosocial work stressors, and the number of anatomical pain regions by gender.
