# Supplementary material for: Longitudinal Associations between Anatomical Regions of Pain and Work Conditions: A Study from The SwePain Cohort
Source: Int J Environ Res Public Health. 2019 Jun 19;16(12):2167. doi: 10.3390/ijerph16122167 (PMC6617203; doi:10.3390/ijerph16122167)
Supplement: Supplementary file 1 [file ijerph-16-02167-s001.pdf]

Table S1. STROBE statement checklist—checklist of items that should be included in reports of observational studies.

	Item No	Recommendation	Page # Line
Title and abstract	1	(a) Indicate the study's design with a commonly used term in the title or the abstract	Cover page #4–5
		(b) Provide in the abstract an informative and balanced summary of what was done and what was found	1 # 3–22
Introduction			
Background/rationale	2	Explain the scientific background and rationale for the investigation being reported	2–3 # 26–54
Objectives	3	State specific objectives, including any prespecified hypotheses	4 #55–64
Methods			
Study design	4	Present key elements of study design early in the paper	3 # 66–72
Setting	5	Describe the setting, locations, and relevant dates, including periods of recruitment, exposure, follow-up, and data collection	3/4 # 66–81
Participants	6	(a) Cohort study—Give the eligibility criteria, and the sources and methods of selection of participants. Describe methods of follow-up<br>Case-control study—Give the eligibility criteria, and the sources and methods of case ascertainment and control selection. Give the rationale for the choice of cases and controls<br>Cross-sectional study—Give the eligibility criteria, and the sources and methods of selection of participants	3/4 # 66–81
		(b) Cohort study—For matched studies, give matching criteria and number of exposed and unexposed<br>Case-control study—For matched studies, give matching criteria and the number of controls per case	Not applicable
Variables	7	Clearly define all outcomes, exposures, predictors, potential confounders, and effect modifiers. Give diagnostic criteria, if applicable	4/6 # 82–141
Data sources/measurement	8*	For each variable of interest, give sources of data and details of methods of assessment (measurement). Describe comparability of assessment methods if there is more than one group	4/6 # 82–141
Bias	9	Describe any efforts to address potential sources of bias	6 # 124–141
Study size	10	Explain how the study size was arrived at	Figure 1
Quantitative variables	11	Explain how quantitative variables were handled in the analyses. If applicable, describe which groupings were chosen and why	4/6 # 82–141
Statistical methods	12	(a) Describe all statistical methods, including those used to control for confounding	6/8 # 142–178
		(b) Describe any methods used to examine subgroups and interactions	7 # 167–171
		(c) Explain how missing data were addressed	
		(d) Cohort study—If applicable, explain how loss to follow-up was addressed<br>Case-control study—If applicable, explain how matching of cases and controls was addressed<br>Cross-sectional study—If applicable, describe analytical methods taking account of sampling strategy	7 # 164–165
		(e) Describe any sensitivity analyses	7 # 167–171
Results			
Participants	13	(a) Report numbers of individuals at each stage of study—e.g., numbers potentially eligible, examined for eligibility, confirmed eligible, included in the study, completing follow-up, and analysed	Figure 1
		(b) Give reasons for non-participation at each stage	8 # 180–188
		(c) Consider use of a flow diagram	Figure 1
Descriptive data	14	(a) Give characteristics of study participants (e.g., demographic, clinical, social) and information on exposures and potential confounders	8 # 180–188 Tables 1,2
		(b) Indicate number of participants with missing data for each variable of interest	Not applicable
		(c) Cohort study—Summarise follow-up time (e.g., average and total amount)	Tables 1,2
Outcome data	15	Cohort study—Report numbers of outcome events or summary measures over time	Tables 1–4

		Case-control study—Report numbers in each exposure category, or summary measures of exposure	
		Cross-sectional study—Report numbers of outcome events or summary measures	
Main results	16	(a) Give unadjusted estimates and, if applicable, confounder-adjusted estimates and their precision (e.g., 95% confidence interval). Make clear which confounders were adjusted for and why they were included	8/10 # 189–220 Tables 3,4
		(b) Report category boundaries when continuous variables were categorized	
		(c) If relevant, consider translating estimates of relative risk into absolute risk for a meaningful time period	
Other analyses	17	Report other analyses done—e.g., analyses of subgroups and interactions, and sensitivity analyses	9/10 # 221–231
Discussion			
Key results	18	Summarise key results with reference to study objectives	10 # 234–246
Limitations	19	Discuss limitations of the study, taking into account sources of potential bias or imprecision. Discuss both direction and magnitude of any potential bias	13 # 298–318
Interpretation	20	Give a cautious overall interpretation of results considering objectives, limitations, multiplicity of analyses, results from similar studies, and other relevant evidence	11/12 # 248–296
Generalisability	21	Discuss the generalisability (external validity) of the study results	13 # 300–303
Other information			
Funding	22	Give the source of funding and the role of the funders for the present study and, if applicable, for the original study on which the present article is based	15 # 339–343

Table S2. Model of changes over time between workload, psychosocial work stressors, and the number of anatomical pain regions by age.

**Outcome and Exposure Variables**	**Crude Models**		**Baseline Adjusted Models ***		**Fully Adjusted Models ****	
	**B (95% CI)**	***p*-Value**	**B (95% CI)**	***p*-Value**	**B (95% CI)**	***p*-Value**
**Age < 50 years**						
**Model of change 1, outcome: change in the number of ARP**						
Predictor: change in mechanical exposure index	0.12 (0.10 to 0.13)	<0.001	0.12 (0.11 to 0.13)	<0.001	0.08 (0.07 to 0.08)	<0.001
Predictor: change in physical exposure index	0.09 (0.07 to 0.10)	<0.001	−0.04 (−0.04 to −0.03)	<0.001	−0.00 (−0.00 to 0.00)	0.220
Predictor: change in job demands	0.57 (0.55 to 0.59)	<0.001	0.31 (0.29 to 0.34)	<0.001	0.01 (−0.01 to 0.04)	0.168
Predictor: change in job control	−0.35 (−0.37 to −0.32)	<0.001	−0.11 (−0.14 to −0.08)	<0.001	0.18 (0.15 to 0.21)	<0.001
Predictor: change in job support	−0.63 (−0.65 to −0.61)	<0.001	−0.41 (−0.44 to −0.38)	<0.001	−0.09 (−0.11 to −0.06)	<0.001
**Age ≥ 50 years**						
**Model of change 1, outcome: change in the number of ARP**						
Predictor: change in mechanical exposure index	0.17 (0.16 to 0.17)	<0.001	0.18 (0.17 to 0.18)	<0.001	0.12 (0.11 to 0.13)	<0.001
Predictor: change in physical exposure index	0.13 (0.12 to 0.14)	<0.001	−0.08 (−0.09 to −0.08)	<0.001	−0.00 (−0.00 to 0.00)	0.278
Predictor: change in job demands	0.71 (0.68 to 0.73)	<0.001	0.28 (0.25 to 0.30)	<0.001	0.03 (−2.17 to 0.06)	0.057
Predictor: change in job control	−0.82 (−0.85 to −0.79)	<0.001	−0.50 (−0.54 to −0.47)	<0.001	−0.07 (−0.10 to −0.03)	<0.001
Predictor: change in job support	−0.89 (−0.92 to −0.86)	<0.001	−0.57 (−0.60 to −0.54)	<0.001	0.01 (−0.02 to 0.05)	0.382
**Age < 50 years**						
**Model of change 2, outcomes: workload and psychosocial work stressors**						
Model of change 2, outcome: change in mechanical exposure index						
Predictor: change in the number of ARP	0.28 (0.27 to 0.29)	<0.001	0.14 (0.13 to 0.14)	<0.001	0.10 (0.09 to 0.11)	<0.001
Model of change 2, outcome: change in physical exposure index						
Predictor: change in the number of ARP	0.13 (0.12 to 0.14)	<0.001	−0.03 (−0.03 to −0.02)	<0.001	−0.00 (−0.00 to 0.00)	0.219
Model of change 2, outcome: change in job demands						
Predictor: change in the number of ARP	0.03 (0.03 to 0.03)	<0.001	0.02 (0.01 to 0.02)	<0.001	0.00 (0.00 to 0.00)	0.169
Model of change 2, outcome: change in job control						
Predictor: change in the number of ARP	−0.01 (−0.02 to −0.01)	<0.001	−0.01 (−0.01 to −0.00)	<0.001	0.01 (0.01 to 0.01)	<0.001
Model of change 2, outcome: change in job support						
Predictor: change in the number of ARP	−0.03 (−0.03 to −0.02)	<0.001	−0.02 (−0.02 to −0.01)	<0.001	−0.01 (−0.01 to −0.01)	<0.001
**Age ≥ 50 years**						
**Model of change 2, outcomes: workload and psychosocial work stressors**						
Model of change 2, outcome: change in mechanical exposure index						
Predictor: change in the number of ARP	0.31 (0.30 to 0.32)	<0.001	0.16 (0.15 to 0.16)	<0.001	0.13 (0.12 to 0.14)	<0.001
Model of change 2, outcome: change in physical exposure index						
Predictor: change in the number of ARP	0.13 (0.12 to 0.13)	<0.001	−0.04 (−0.05 to −0.01)	<0.001	−0.00 (−0.01 to 0.01)	0.277
Model of change 2, outcome: change in job demands						
Predictor: change in the number of ARP	0.03 (0.03 to 0.03)	<0.001	−0.02 (−0.02 to −0.01)	<0.001	−0.01 (−0.01 to 0.01)	0.810
Model of change 2, outcome: change in job control						
Predictor: change in the number of ARP	−0.02(−0.02 to −0.02)	<0.001	−0.01 (−0.01 to −0.01)	<0.001	−0.01 (−0.01 to −0.00)	<0.001
Model of change 2, outcome: change in job support						
Predictor: change in the number of ARP	−0.03 (−0.03 to −0.02)	<0.001	−0.01 (−0.02 to −0.01)	<0.001	0.00 (−0.01 to 0.01)	0.382

ARP = anatomical regions with pain; B = regression coefficients; CI = confidence intervals. * Model adjusted for changes in workload, psychosocial work stressors, and number of ARP; ** Model adjusted for time-independent gender and time-depended changes in, education, smoking, alcohol intake, psychical activity, distress, and changes in workload, psychosocial work stressors, and the number of ARP.

Table S3. Model of changes over time between workload, psychosocial work stressors, and the number of anatomical pain regions by gender.

Outcome and Exposure Variables	Crude Models		Baseline Adjusted Models *		Fully Adjusted Models **	
	B (95% CI)	*p*-Value	B (95% CI)	*p*-Value	B (95% CI)	*p*-Value
**Men**						
**Model of change 1, outcome: change in the number of ARP**						
Predictor: change in mechanical exposure index	0.11 (0.11 to 0.12)	<0.001	0.11 (0.10 to 0.11)	<0.001	0.08 (0.08 to 0.09)	<0.001
Predictor: change in physical exposure index	0.12 (0.11 to 0.12)	<0.001	−0.02 (−0.02 to −0.01)	<0.001	−0.00 (−0.01 to 0.02)	0.235
Predictor: change in job demands	0.53 (0.51 to 0.55)	<0.001	0.22 (0.20 to 0.24)	<0.001	0.03 (0.01 to 0.05)	0.002
Predictor: change in job control	−0.26(−0.28 to −0.24)	<0.001	−0.01 (−0.03 to 0.11)	0.355	0.04 (0.02 to 0.07)	0.001
Predictor: change in job support	−0.59 (−0.61 to −0.57)	<0.001	−0.38 (−0.40 to −0.36)	<0.001	−0.02 (−0.04 to 0.01)	0.183
**Women**						
**Model of change 1, outcome: change in the number of ARP**						
Predictor: change in mechanical exposure index	0.19 (0.18 to 0.19)	<0.001	0.18 (0.18 to 0.19)	<0.001	0.13 (0.12 to 0.14)	<0.001
Predictor: change in physical exposure index	0.14 (0.13 to 0.15)	<0.001	−0.05 (−0.06 to −0.04)	0.006	0.00 (−0.00 to 0.01)	0.603
Predictor: change in job demands	0.63 (0.60 to 0.65)	<0.001	0.18 (0.16 to 0.21)	<0.001	−0.04 (−0.07 to −0.01)	0.015
Predictor: change in job control	−0.54 (−0.56 to −0.50)	<0.001	−0.15 (−0.18 to −0.11)	<0.001	0.00 (−0.04 to 0.04)	0.985
Predictor: change in job support	−0.82 (−0.44 to −0.38)	<0.001	−0.61 (−0.65 to −0.58)	<0.001	−0.06 (−0.09 to −0.02)	0.002
**Men**						
**Model of change 2, outcomes: workload and psychosocial work stressors**						
Model of change 2, outcome: change in mechanical exposure index						
Predictor: change in the number of ARP	0.48 (0.47 to 0.49)	<0.001	0.20 (0.19 to 0.21)	<0.001	0.18 (0.17 to 0.19)	<0.001
Model of change 2, outcome: change in physical exposure index						
Predictor: change in the number of ARP	0.25 (0.24 to 0.25)	<0.001	−0.02 (−0.02 to −0.01)	<0.001	−0.01 (−0.01 to 0.01)	0.235
Model of change 2, outcome: change in job demands						
Predictor: change in the number of ARP	0.04 (0.03 to 0.04)	<0.001	0.02 (0.01 to 0.02)	<0.001	0.00 (0.00 to 0.00)	0.255
Model of change 2, outcome: change in job control						
Predictor: change in the number of ARP	−0.01 (−0.02 to −0.01)	<0.001	−0.00 (−0.00 to 0.00)	0.355	0.00 (0.00 to 0.00)	0.001
Model of change 2, outcome: change in job support						
Predictor: change in the number of ARP	−0.04 (−0.04 to −0.03)	<0.001	−0.02 (−0.02 to −0.01)	<0.001	−0.00 (−0.00 to 0.00)	0.184
**Women**						
**Model of change 2, outcomes: workload and psychosocial work stressors**						
Model of change 2, outcome: change in mechanical exposure index						
Predictor: change in the number of ARP	0.23 (0.23 to 0.24)	<0.001	0.21 (0.15 to 0.22)	<0.001	0.19 (0.18 to 0.20)	<0.001
Model of change 2, outcome: change in physical exposure index						
Predictor: change in the number of ARP	0.11 (0.10 to 0.11)	<0.001	−0.02 (−0.03 to −0.02)	<0.001	0.00 (−0.00 to 0.01)	0.603
Model of change 2, outcome: change in job demands						
Predictor: change in the number of ARP	0.02 (0.02 to 0.03)	<0.001	0.01 (0.00 to 0.01)	<0.001	−0.01 (−0.01 to −0.00)	0.015
Model of change 2, outcome: change in job control						
Predictor: change in the number of ARP	−0.01 (−0.02 to −0.01)	<0.001	−0.01 (−0.01 to −0.01)	<0.001	−0.00 (−0.00 to 0.00)	0.985
Model of change 2, outcome: change in job support						
Predictor: change in the number of ARP	−0.02 (−0.03 to −0.02)	<0.001	−0.01 (−0.02 to −0.01)	<0.001	−0.01 (−0.01 to −0.00)	0.002

ARP = anatomical regions with pain; B = regression coefficients; CI = confidence intervals. * Model adjusted for changes in workload, psychosocial work stressors, and number of ARP; ** Model adjusted for time-depended changes in age, education, smoking, alcohol intake, psychical activity, distress, and changes in workload, psychosocial work stressors, and the number of ARP.
